# Application of Ultrasound Elastography in Assessing Portal Hypertension

**DOI:** 10.3390/diagnostics12102373

**Published:** 2022-09-29

**Authors:** Man Zhang, Hongyu Jin, Jiazhi Cao, Ruyu Ren, Menglu Jia, Yi Yang, Xinyi Li, Ming Chen, Shen Li, Libin Huang, Wenwu Ling

**Affiliations:** 1 West China School of Medicine, West China Hospital, Sichuan University, Chengdu 610041, China; 2 Department of Liver Surgery & Liver Transplantation, State Key Laboratory of Biotherapy and Cancer Center, West China Hospital, Sichuan University and Collaborative Innovation Center of Biotherapy, Chengdu 610041, China; 3 Department of Ultrasound, West China Hospital, Sichuan University, Chengdu 610041, China; 4 Department of Geriatrics, Peking University Health Science Center, Beijing 100191, China; 5 Department of Gastroenterology, West China Hospital, Sichuan University, Chengdu 610041, China

**Keywords:** ultrasound elastography, liver stiffness measurement, spleen stiffness measurement, portal hypertension, gastro-esophageal varices

## Abstract

Portal hypertension is a common manifestation in late-to-end-stage liver diseases and can cause severe complications such as ascites, hepatic encephalopathy, etc. However, an early diagnosis of portal hypertension is often difficult as it can be asymptomatic. Though the gold standard to diagnose portal hypertension is hepatic vein catheterization, ultrasound elastography is regarded as a noninvasive alternative that can be used to accurately predict portal hypertension and a few further complications such as gastro-esophageal varices. Since ultrasound elastography is available in most medical centers, and is cheaper and noninvasive, studying its function in predicting portal hypertension is of paramount importance. Therefore, this review generalized the results of recently published articles in order to establish the indicators that were related to diagnostic and prediction efficiency. Our study found that various technologies of ultrasound elastography could be used to predict portal hypertension with satisfactory diagnostic sensitivity, specificity, accuracy, and AUC. Meanwhile, we also recognized similar diagnostic efficiency of ultrasound elastography in gastro-esophageal varices.

## 1. Introduction

Portal hypertension is a common disease manifesting in a large number of liver diseases, especially in liver cirrhosis. It induces a number of life-threatening complications, including esophageal and gastric bleeding, hepatic encephalopathy, liver failure, ascites, spontaneous bacterial peritonitis, hepato-renal syndrome, etc. [1,2]. Portal hypertension tends to be asymptomatic and develops slowly during the early phrase, thus patients are more likely to suffer from many complications simultaneously once the symptoms occur [3]. Etiologically, portal hypertension can be caused by several conditions; therefore, it accompanies over half of the population with liver diseases. Similar to systemic hypertension, portal hypertension may occur when there is a sudden or substantial augment in hepatic bloodstream, which appears to increase the intra-vascular pressure or impeding to intrahepatic bloodstream [4]. For patients with primary liver diseases, an increase in impeding to intrahepatic bloodstream, due to structural changes in liver parenchyma between hepatic cells, is the most familiar driving factor for portal hypertension [5]. These hepatic structural changes can result from a variety of chronic liver diseases, such as a chronic infection of the hepatitis B virus (HBV), alcoholic liver disease, fatty liver diseases, etc., which maintain a comparatively high prevalence rate [6]. In addition to the aforementioned chronic liver diseases, liver vascular abnormalities include hepatic vein thrombosis, the obstruction of extrahepatic veins, or other idiopathic causes [7]. As such, generally speaking, there exists a substantial number of diseases and conditions that may lead to portal hypertension.

The developing course of portal hypertension is generally quite slow and may remain undetermined for many years. The process of portal hypertension can be divided into asymptomatic and symptomatic phases. During the first stage, the patient may feel nothing except for slight fatigue or abdominal discomfort, which is likely to be ignored [8]. Therefore, patients mostly go undiagnosed unless they pay regular visits to family physicians or regular medical examinations are arranged, which is not viable for most of the population [9]. However, it is worth noting that some of the results from medical examinations can be abnormal during this stage. These include abnormal liver function, abnormal routine blood examination (thrombocytopenia), and changes in the stiffness of the liver which can be found during ultrasound, computed tomography (CT), or magnetic resonance imaging (MRI) examinations despite the patient being asymptomatic [10]. Regrettably, many of these abnormalities in the screening tests lack specificity and thus are likely to be overlooked. Luckily, in view of the high prevalence of chronic liver diseases and liver vascular diseases, basic screening tests, including routine blood, liver function, and ultrasound examinations, still remain the cornerstone of regular medical examinations [11]. In addition, since splenomegaly is a common complication of portal hypertension, accidental findings of splenomegaly can help to facilitate diagnosing portal hypertension [12]. Furthermore, portal hypertension tends to develop quite fast once the patient becomes symptomatic.

Normally, the majority of portal hypertension cases are correlated with liver cirrhosis, which itself is a manifestation of end-term liver disease. Thus, several invasive techniques are often used to directly, or indirectly, determine the existence of portal hypertension. The golden standard to diagnose hypertension when hepatic venous pressure gradient (HVPG) >5 mmHg is via catheterization into the hepatic vein in order to measure HVPG. Furthermore, this technique is commonly used in cirrhotic portal hypertension [13]. Other than this, endoscopy helps to determine the existence of portal hypertension by the detection of indirect signs, like gastro-esophageal varices or the rupture of the gastro-esophageal varicose veins, which can also help classify the lesion for its risk of rupture [14]. In addition, pathological findings such as intrahepatic shunt formation, intrahepatic neo-angiogenesis, liver sinusoids capillarization, and abnormal fibrogenesis can also help to diagnose portal hypertension after a liver biopsy or hepatectomy [15]. However, for patients with a comparatively decompensated blood coagulation ability, the diagnostic risk is relatively high since these methods are invasive. In addition, hepatic vein catheterization, endoscopy, and liver biopsy (or hepatectomy) are not frequently prescribed or are not available in smaller medical centers. Therefore, looking for noninvasive alternatives is key to increase early diagnosis rate and efficacy in treating portal hypertension.

Ultrasound elastography is regarded as a safe and accurate method to quantitatively measure the stiffness of the liver and subsequently judge the existence of portal hypertension [16]. Normally, deformation occurs when biological tissues become pressed, and different tissues manifest different degrees of deformation because of their differences in elasticity coefficients. Therefore, if an external force is imposed upon them, or an alternating vibration is applied, tissues will respond with different signals. If we collect these signals and analyze them with a combined autocorrelation method, we can evaluate the hardness of the tissues. Additionally, if we use colors to define these signals and manifest them on a screen, physicians can observe the tissues’ hardness intuitively. The older, previous version of ultrasound elastography is called vibration-controlled transient elastography [17]. Under this mechanism, a vibration is made and transmitted to the liver by a specific probe placed on the skin of the intercostal space on the right side, and the responsive shear-wave distortion is measured by a specific beam on the probe [18]. However, this method gives the operator limited control in terms of the position to place the probe. With the advancement in physics and ultrasound technology, point shear-wave elastography (pSWE) and two-dimensional shear-wave elastography (2D-SWE) have been developed. These methods allow comparatively free observations over larger proportions of the liver and are made possible by using more focused short-duration and high-intensity acoustic pulses [19]. With this technology, simultaneous color-coded observations of liver on the screen of the ultrasound device, as well as quantitative measurement is possible. Moreover, SWE is intended to be less operator-dependent compared with transient elastography, therefore it maintains the potential of promotion, especially to health care providers in remote areas, since it is easier for physicians to learn. Nowadays, either pSWE or 2D-SWE are available in almost all ultrasonic devices [20].

Since a large proportion of patients with portal hypertension remain asymptomatic and therefore are not likely to undergo invasive diagnosing techniques, noninvasive diagnosing methods are thus paid special attention to. Among them, as a cheap, time saving, and widely applied method, great expectations are therefore placed on ultrasound elastography. As such, this mini review discussed the rationale, diagnostic accuracy, and limitations of ultrasound elastography in diagnosing portal hypertension and its potential role in predicting gastro-esophageal varices. In addition, the subsidiarity of spleen stiffness measurement (SSM) in helping determine portal hypertension is also discussed.

## 2. Diagnosis of Portal Hypertension via Liver Stiffness Measurement (LSM)

As a major method of ultrasound elastography, LSM is believed to have the capacity to indicate the degree of fibrosis of the liver. In 2022, Semmler et al. studied the diagnostic efficiency of LSM/platelet count (PLT) in clinically significant portal hypertension in patients who have received successful antiviral therapy after HCV infection. Their study found the condition that LSM < 12 kPa and PLT > 150 g/L after HCV treatment could rule out the possibility of clinically significant portal hypertension with a sensitivity of 99.2% [21]. A multicenter study in Switzerland, France, and Spain, which included 155 patients with porto-sinusoidal vascular liver disease (PSVD) and 273 patients with liver cirrhosis, pointed out that LSM-using transient elastography (TE-LSM) could help to judge the presence of portal hypertension. Moreover, a cut-off value of 10 kPa can maintain a high specificity of up to 97% in diagnosing PSVD and a cut-off value of 20 kPa can maintain a high specificity of up to 94% in ruling out PSVD [22]. In 2020, a multicenter cohort study was carried out to look for correlations of LSM results with standard biochemical results with regard to liver function and the correlation between LSM and portal hypertension in children with liver diseases. A total of 550 children were included and LSM was carried out. The study indicated a positive correlation between several biochemical indicators (total bilirubin, ALT, AST, gamma-glutamyltranspeptidase (GGT), international normalized ratio (INR), GGT to platelet ratio (GPR), AST to platelet ratio, spleen size, pediatric end-stage liver disease score, and the existence of portal hypertension. Meanwhile, the study also pointed out a more detailed correlation of LSM and portal hypertension grades, indicating the important role of LSM in classifying both the existence and degree of portal hypertension [23]. Another study published in 2020 included 140 patients with clinically significant portal hypertension (CSPH) and investigated the diagnostic performance of LSM of portal hypertension. The study pointed out that LSM was significantly correlated with the occurrence of portal hypertension. In addition, the study also compared the diagnostic efficacy of LSM with the liver surface nodularity (LSN) and found they had quite similar diagnostic efficiency. Moreover, their group found that combining LSM and LSN could increase the detection rate of portal hypertension [24]. Another study in 2020 compared the diagnostic performance of portal hypertension between vibration-controlled transient elastography (VCTE) and 2D-SWE. By recruiting 127 patients with chronic liver diseases and determining the presence of portal hypertension by hepatic vein catheterization, the study found that 2D-SWE was significantly efficient to detect portal hypertension, in particular when HVPG < 10 mmHg. When the cut-off value was 11.3 kPa, the detection rate of clinically significant portal hypertension could reach 0.91. In addition, the study indicated there was no significant difference between the diagnostic efficacy of VCTE and 2D-SWE [25]. A study in 2019 investigated the role of LSM in determining the presence of portal hypertension in Budd–Chiari Syndrome (BCS) and concluded that patients with BCS often manifested extremely high LSM (75 kPa) and that the high LSM could persist until transjugular intrahepatic portosystemic shunt (TIPS) treatment was conducted [26]. A systematic review and meta-analysis in 2020 investigated the efficiency of transient elastography-based LSM for diagnosing portal hypertension in 679 patients who had alcoholic liver diseases. The study concluded that the sensitivity and specificity for diagnosing portal hypertension using LSM were 0.89 and 0.71, respectively, when the cut-off value was 21.8 kPa; further, the sensitivity and specificity for diagnosing severe portal hypertension (SPH) using LSM were 0.88 and 0.74, respectively, when the cut-off value was 29.1 kPa [27]. A 2020 study investigated the use of 2D-SWE in treating HBV-related liver cirrhosis and found that the 2D-SWE value was highly correlated with HVPG in portal hypertension and the severe portal hypertension group. In addition, a sensitivity and specificity of 0.78 and 0.72 in diagnosing portal hypertension (cut-off value of 16.1 kPa) and 0.81 and 0.79 in diagnosing severe portal hypertension (cut-off value of 23.5 kPa) was found [28]. Another study focused on the role of LSM in diagnosing portal hypertension in patients with liver cirrhosis when combined with large esophageal varices. After enrolling 99 cirrhotic patients, the study calculated the diagnostic accuracy, sensitivity, and specificity of 0.75, 0.78, and 0.54, respectively, with a cut-off value of 16.0 kPa [29]. In 2018, 910 patients were enrolled in a systematic review and meta-analysis with an aim to investigate the diagnostic efficacy of LSM via different cut-off values to define clinically significant portal hypertension in chronic viral liver diseases. The study found that the sensitivity and specificity values could reach 0.96 and 0.60, respectively, when the cut-off value was 13.6 kPa; 0.85 and 0.80 when the cut-off value was 18 kPa; and 0.74 and 0.94 when the cut-off value was 22 kPa. In addition, the study pointed out that LSM could reach its best diagnostic performance when the cut-off value was around 22 kPa [30]. In the same year, another study recruiting 191 patients found that the sensitivity and specificity of LSM could reach 0.95 and 0.52, respectively [31]. In 2017, a study claimed that the sensitivity and specificity of LSM to diagnose clinically significant portal hypertension in children were 0.875 and 0.84, respectively, when the cut-off value was 18.4 kPa. They also confirmed a solid correlation between LSM and HVPG [32]. Before 2017, there were also a number of studies focusing on the diagnostic role of LSM in clinically significant portal hypertension. The results of these studies are shown in Table 1 [22,23,24,25,27,28,32,33,34,35,36,37,38,39,40,41,42,43,44].

## 3. Limitations of LSM

According to many published and ongoing studies, LSM could be regarded as a potential noninvasive method to recognize portal hypertension with a satisfactory diagnostic accuracy, sensitivity, and specificity. Meanwhile, as a cheap and widely applied noninvasive method, LSM could also be used in disease-screening procedures among people with a high risk of portal hypertension. However, there also existed certain potential limitations in defining portal hypertension via LSM. First of all, the mechanism of applying LSM as a potential way to describe portal hypertension was that the fibrotic essence of a liver could be detected by ultrasound techniques. However, even if a fibrotic change in the liver was the number one reason to cause abnormality in liver mechanical properties, there were also a number of other reasons that induced change in liver mechanical property, such as inflammation, venous congestion, infiltrative liver disease, cholestasis, etc. [45,46]. As a result, false positive results might appear in these groups of patients. Secondly, although studies in recent years have suggested that many methods of ultrasound elastography maintained relatively satisfactory stability, differences of results by different kinds of ultrasound elastography were also frequently noted and observed [47,48]. Consequently, disciplines and experiences targeted to a specific technique of ultrasound elastography or even a specific kind of machine should be established.

## 4. Diagnostic Role of Splenic Stiffness Measurement (SSM) in Portal Hypertension

Splenomegaly is observed in a large fraction of patients with portal hypertension. This can be attributed to passive congestion of the spleen aiming to increase arterial blood inflow. Meanwhile, splenomegaly can also result from an abnormally active proliferation of the splenic lymphoid tissue, an abnormally enhanced angiogenesis, as well as fibrosis [49]. Enhanced fibrosis of the spleen could change its physical property, such as stiffness, which could be described by ultrasound stiffness evaluation. Moreover, studies have shown that splenic vascular resistance caused by increased angiogenesis could be observed by Doppler pulsatility and resistance indicators, the value of which were related to the extent of portal hypertension as well as the severity of the patients’ syndromes and complications [50]. Therefore, physicians have speculated whether SSM could be an alternative, or an accessory, method to noninvasively predict portal hypertension. In this regard, studies in recent years have provided valuable evidence. In 2021, a review concluded that SSM correlated well with HVPG. Additionally, the study also pointed out that clinically significant portal hypertension was likely to exist when spleen stiffness was higher than 40–45 kPa [51]. Another cohort study of 140 patients after HCV eradication also pointed out that SSM values were correlated with incidences of portal hypertension. More importantly, this study also found that SSM could help predict the possibility of developing hepatocellular carcinoma (HCC) in this group of patients, where the best cut-off value is 42 kPa [52]. A systematic review and meta-analysis, published in 2020 and including 32 studies with 3952 patients, found that the sensitivity and specificity of SSM to predict clinically significant portal hypertension were determined at 0.85 and 0.86, respectively. The study also concluded that the sensitivity and specificity of the SSM to predict severe portal hypertension were 0.84 and 0.84, respectively. In addition, the study claimed that SSM could predict gastroesophageal varices with a sensitivity and specificity of 0.90 and 0.73 [53], respectively. Another study aimed to find potential predictive value of SSM for decompensating events in patients with chronic liver diseases. The study also found that the patients with decompensating events would show a comparatively higher SSM value (44 kPa vs. 30 kPa, *p* < 0.001), higher spleen diameter (14 cm vs. 12 cm, *p* = 0.043), and a lower platelet count (94.5 g/L vs. 121.5 g/L, *p* < 0.001) [54]. In 2020, a study recruited children with obstruction in extrahepatic portal vein, and divided the patients into three groups (group A: patients with extrahepatic portal hypertension without spontaneous portosystemic shunts in large scale; group B: patients with extrahepatic portal hypertension with spontaneous portosystemic shunts in large scale; group C: patients with extrahepatic portal vein obstruction after surgical portosystemic shunts). This study established that SSM values were significantly different in group A (70 ± 4.64 kPa) when compared to those in group B (37.04 ± 4.62 kPa) and group C (26.3 ± 2.9 kPa). In addition, the study claimed that SSM could be an important way of achieving a long-term follow up in patients with obstruction in extrahepatic portal vein after surgery and therefore assist with predictions of esophageal varices [55]. In 2019, a study recruited patients with human immunodeficiency virus (HIV) and noncirrhotic portal hypertension (NCPH) and divided them into three groups (group A: patients with HIV and known NCPH; group B: patients with HIV with past exposure to didanosine without known liver diseases or NCPH; and group C: patients with HIV without either exposure to didanosine or liver diseases or NCPH). The study found that the SSM score was highly related to the LSM score and an elevated SSM score could predict NCPH. In addition, the diagnostic sensitivity, specificity, positive predictive value, negative predictive value, positive likelihood ratio, and negative likelihood ratio, were 0.91, 0.93, 0.91, 0.93, 12.73, and 0.10, respectively, when the cut-off value was 25.4 kPa [56]. A study investigating the use of SSM in diagnosing portal hypertension in patients with HBV-related cirrhosis found high sensitivity (0.85) and specificity (0.79) values with the best cut-off value of 25.3 kPa for clinically significant portal hypertension along with values of 0.74 and 0.70 with the best cut-off value of 33.4 kPa for severe portal hypertension [28]. In 2018, a group of physicians targeted the surveillance function of SSM in portal hypertension in patients with HCV infection and successfully treated by the application of interferon. Their data showed that SSM score could be an efficient marker in long-term follow up with respect to observing portal hypertension in patients with HCV after antiviral therapy [57]. Ninety-nine patients with liver cirrhosis and concomitant large esophageal varices were enrolled in a study to study the diagnostic efficacy of SSM in portal hypertension. The study found that the diagnostic accuracy, sensitivity, specificity, positive predictive value, and negative predictive value of 0.79, 0.76, 1.00, 1.00, and 0.38, respectively, with a cut-off value of 48.9 kPa [29]. A systematic review and meta-analysis including nine studies in 2018 confirmed the correlation between the SSM value and HVPG, and also found the summary coefficient to be 0.72. In addition, the study found the sensitivity, specificity, AUC, and DOR of 0.88, 0.84, 0.92, and 38 when detecting clinically significant portal hypertension [58]. In addition to the aforementioned studies, a couple of other studies have also mentioned the diagnostic efficiency of SSM in describing portal hypertension. The details are provided in Table 2 [28,29,33,34,35,36,44,53,56,59,60,61,62,63,64,65,66,67,68].

## 5. Description of Gastro-Esophageal Varices via LSM or SSM

Gastro-esophageal varices are one of the most common accompanied situations of end-stage liver diseases, which is a direct result of portal hypertension. Since many studies have recognized the potential role of LSM and SSM in defining portal hypertension, a number of scientists have speculated whether LSM and SSM could predict gastro-esophageal varices. In 2021, 661 patients with compensated advanced chronic liver disease (cACLD) receiving LSM in the form of 2D-SWE were enrolled in a study in order to investigate whether 2D-SWE could predict gastro-esophageal varices. These patients also received an endoscopy to confirm the diagnostic efficacy of LSM. Their results showed that platelet count, albumin level, and LSM scores were all independent predictive factors for varices. They also found that the screening criteria of LSM < 16 kPa and platelet count > 100 × 10^9^/L could reduce the necessity for an endoscopy by 30.4–34.6% [69]. Another study, published in 2021, recruited 107 patients with compensated liver cirrhosis to compare pSWE and 2D-SWE of SSM in their potential to predict gastro-esophageal varices. The results showed that the optimal cut-off value for SSM via pSWE and 2D-SWE were 13.2 kPa and 2.91 m/s, respectively. The study also showed that SSM score was a reliable auxiliary marker with satisfactory workability, serviceability and predictability. However, there did not exist a significant difference in the diagnostic efficacy of p-SWE and 2D-SWE [70]. Moreover, another study recruited 102 patients with chronic liver diseases after treatment of TIPS, to investigate the role of SSM in the long-term follow up of gastro-esophageal varices. Regarding short-term follow up, the AUCs of SSM were 0.585 (mild varices), 0.655 (moderate varices), and 0.739 (severe varices). Concerning long-term follow up, the AUCs of SSM were 0.778 (mild varices), 0.82 (moderate varices), and 0.824 (severe varices) [71]. One hundred and seven children were recruited to investigate the relative efficacy of LSM and SSM in predicting gastro-esophageal varices. Among them, 52 children were blank controls and the other 55 children had pediatric extrahepatic portal vein obstruction (EHPVO). The study found that there was no difference in SSM score between EHPVO patients with gastro-esophageal varices and those without. While SSM seemed to be indifferent between the two groups, LSM was higher in EHPVO patients with gastro-esophageal varices than those without (1.19 vs. 1.10, *p* = 0.003) [72]. Another study recruited 97 patients with HCV related liver cirrhosis who were treated with orally-taken antiviral agents, in order to investigate the predictive ability of esophageal varices via LSM, SSM, and the liver stiffness–spleen diameter to platelet ratio score. The study found that LSM (12.2 vs. 16, *p* = 0.02), SSM (39.4 vs. 46.05, *p* = 0.04), and the liver stiffness-spleen diameter to platelet ratio score (1102.19 vs. 829.7, *p* = 0.04) were conspicuously correlated in patients both with and without esophageal varices [73]. A study published in 2021 provided a calculated score for forecasting the situations of varices which were high in risk in patients diagnosed with compensated liver cirrhosis by performing a multiple regression analysis in 124 patients. The study established a novel combined score (=0.053 × SSM + 0.054 × LSM + 0.059 × spleen size) that could predict high-risk varices when the score > 0.034 (AUC = 0.93) [74].

## 6. Conclusions

Portal hypertension is the most common manifestation in a variety of chronic liver diseases. Thus, early and accurate screening and diagnosis of portal hypertension is extremely important in decreasing the proportion of end-stage liver diseases. However, early stages of portal hypertension are mostly asymptomatic, which discourages patients to pay clinical visit. In addition, the golden standard for diagnosing portal hypertension is catheterization into the hepatic vein, which is an invasive, expensive technique and therefore is hard to be promoted widely. As a comparatively cheap, noninvasive, and widely applied technology, ultrasound elastography has been considered as a potential alternative to predict portal hypertension. We performed this review in order to more profoundly evaluate the predictive and diagnostic value of ultrasound elastography. After reviewing recently published articles, our study found that various techniques of ultrasound elastography including TE-LSM, TE-SSM, pSWE (LSM), pSWE (SSM), 2D-SWE (LSM), and 2D-SWE (SSM) could predict portal hypertension noninvasively with satisfactory diagnostic sensitivity, specificity, accuracy, and AUC. Moreover, our study revealed that other than portal hypertension, ultrasound elastography could also be used to predict gastro-esophageal varices with high diagnostic sensitivity, specificity, accuracy, and AUC. In addition to ultrasound elastography, there are other ways of predicting portal hypertension noninvasively, such as abdominal MRI, CT, contrast-enhanced ultrasonography, etc. In 2021, an international multicenter study, which included 149 patients, concluded that by using a CHESS-DIS score, non-contrast-enhanced abdominal MRI had the potential to detect portal hypertension with an AUC of 0.81 (training cohort) and 0.9 (validation cohort) [75]. Another study found that the ΔT1 value of the liver and spleen, along with the extracellular volume fraction of the spleen, could be a predictor of portal hypertension during an enhanced MRI of Gd-EOB-DTPA [76]. However, since the MRI examination time was much longer and the preparation was more difficult and complicated, few patients underwent MRI at the very beginning. A study recruiting 131 patients concluded that contrast-enhanced ultrasonography could discover portal hypertension with a sensitivity and specificity of up to 88% and 63%, respectively [77]. In 2020, Gupta I et al. found that a subharmonic-aided pressure estimation under contrast-enhanced ultrasonography had a relatively satisfactory sensitivity and specificity of 90% and 80% [78], respectively. Recently, contrast-enhanced ultrasonography has gained attention and popularity, owing to its reasonable cost and convenience.

## Figures and Tables

**Table 1:** Basic characteristics and diagnostic efficacy of LSM in assessing portal hypertension.

First Author	Year	Country	Type of Study	Number of Patients	Disease	Diagnostic Method	Sensitivity	Specificity	Accuracy	AUC	Optimal Cut-Off Value	Correlation Coefficient
Laure Elkrief [22]	2021	Switzerland, France, Spain,	Retrospective	273 patients	Compensated liver cirrhosis	TE-LSM	0.94	0.97	0.84	0.93	20 kPa for best sensitivity and 10 kPa for best specificity	NA
Benjamin L. Shneider [23]	2020	USA, Canada	Prospective	550 patients	Pediatric cholestatic liver disease	TE-LSM	NA	NA	NA	NA	NA	NA
Alexandra Souhami [24]	2020	France	Retrospective	140 patients	Liver cirrhosis	TE-LSM	NA	NA	NA	0.83 for CSPH	13.6 kPa (rule-out); 21 kPa (rule-in)	0.75
Horia Stefanescu [25]	2020	Romania	Prospective	127 patients	Chronic liver disease	2D-SWE (LSM)	0.95 with cut-off value of 9 kPa	0.95 with cut-off value of 13 kPa	0.845 with cut-off value of 11.3 kPa	NA	13.6 kPa	NA
Jinzhen Song [27]	2020	China	Systematic review and meta-analysis	9 studies, 679 patients	Chronic liver disease	TE-LSM	0.89 for CSPH; 0.88 for SPH	0.71 for CSPH; 0.74 for SPH	NA	NA	21.8 kPa for CSPH; 29.1 kPa for SPH	NA
Jinzhen Song [30]	2018	China	Systematic review and meta-analysis	11 studies, 910 patients	Chronic viral liver disease	TE-LSM	0.96 with cut-off value of 13.6 kPa; 0.85 with cut-off value of 18 kPa; 0.74 when cut-off value of 22 kPa	0.60 with cut-off value of 13.6 kPa; 0.80 with cut-off value of 18 kPa; 0.94 when cut-off value of 22 kPa	NA	NA	17.6 kPa	NA
Hee Mang Yoon [32]	2017	Korea	Retrospective	32 patients	Chronic liver disease	SWE (LSM)	0.875	0.84	NA	0.915	18.4 kPa	NA
Romanas Zykus [33]	2015	Lithuania	Prospective	107 patients	Chronic liver disease	TE-SSM	0.88 for CSPH; 0.828 for SPH	0.875 for CSPH; 0.800 for SPH	0.887 for CSPH; 0.831 for SPH	NA	47.6 kPa for CSPH; 50.7 kPa for SPH	NA
Antonio Colecchia [34]	2012	Italy	Prospective	100 patients	HCV related liver cirrhosis	TE-LSM	0.954 for CSPH; 0.981 for SPH	0.686 for CSPH; 0.630 for SPH	NA	NA	16 kPa for CSPH; 16.4 kPa for SPH	NA
D Attia [35]	2015	Germany	Cross-sectional	94 patients	Progressive chronic liver disease	LSM	0.97 for CSPH; 0.93 for SPH	0.89 for CSPH; 0.73 for SPH	NA	0.929 for CSPH; 0.872 for SPH	2.17 m/s for CSPH; 2.54 m/s for SPH	NA
Bogdan Procopet [36]	2015	Spain, Canada	Prospective	88 patients	Compensated liver cirrhosis	2D-SWE (LSM)	0.808	0.821	NA	0.858	17 kPa	NA
Chul Min Lee [37]	2016	Korea	Retrospective	47 patients	Liver cirrhosis resulting from alcohol, HBV, HCV, etc.	SWE (LSM)	0.74	0.83	NA	0.75	19.7 kPa	0.516
Philipp Schwabl [38]	2015	Austria	Retrospective	278 patients	Chronic liver disease	TE-LSM	0.936	0.87	0.889	0.957	16.1 kPa	0.836
Elba Llop [39]	2017	Spain	Retrospective	442 patients	Compensated advanced chronic liver disease	TE-LSM	NA	NA	0.714	NA	28 kPa	NA
M Lemoine [40]	2008	France	Retrospective	44 patients	HCV or alcohol related liver cirrhosis	TE-LSM	0.55	0.90	NA	NA	22.0 kPa	NA
Francesco Vizzutti [41]	2007	Italy	Retrospective	61 patients	HCV related chronic liver disease	TE-LSM	0.97 for CSPH; 0.94 for SPH	NA	NA	0.99 for CSPH; 0.92 for SPH	13.6 kPa for CSPH; 17.6 kPa for SPH	0.81 for CSPH; 0.91 for SPH
M Sanchez-Conde [42]	2011	Spain	Prospective	38 patients	HIV/HCV coinfected with chronic liver disease	TE-LSM	0.9286 for CSPH; 0.8261 for SPH	0.50 for CSPH; 0.6667 for SPH	NA	0.80 for CSPH; 0.80 for SPH	14 kPa for CSPH; 23 kPa for SPH	0.46
Thomas Reiberger [43]	2012	Austria	Prospective	794 patients	Chronic liver damage	TE-LSM	0.956	0.667	NA	0.794	8 kPa	0.799
Praveen Sharma [44]	2013	India	Prospective	270 patients	Liver cirrhosis	TE-SSM	0.91 for esophageal varices	0.72 for esophageal varices	0.86 for esophageal varices	NA	27.3kPa	NA

LSM: liver stiffness measurement; TE: transient elastography; CSHP: clinically significant portal hypertension; SPH: severe portal hypertension; SWE: shear-wave elastography; HBV: hepatitis B virus; HCV: hepatitis C virus; HIV: human immunodeficiency virus; AUC: area under curve.

**Table 2:** Basic characteristics and diagnostic efficacy of SSM in assessing portal hypertension and gastro-esophageal varices.

First Author	Year	Country	Type of Study	Number of Patients	Disease	Diagnostic Method	Sensitivity	Specificity	Accuracy	AUC	Optimal Cut-Off Value	Correlation Coefficient
Yuli Zhu [28]	2019	China	Retrospective	104 patients	HBV related liver cirrhosis	2D-SWE (SSM)	0.85 for CSPH; 0.79 for SPH	0.74 for CSPH; 0.70 for SPH	NA	0.81	25.3 kPa for CSPH; 33.4 kPa for SPH	NA
Yujen Tseng [29]	2018	China	Cross-sectional	99 patients	Liver cirrhosis	TE-SSM	0.76	1.00	0.79	0.91	48.9 kPa	NA
Romanas Zykus [33]	2015	Lithuania	Prospective	107 patients	Chronic liver disease	TE-SSM	0.773 for CSPH; 0.781 for SPH	0.792 for CSPH; 0.771 for SPH	0.777 for CSPH and SPH	NA	47.6 kPa for CSPH; 50.7 kPa for SPH	NA
Antonio Colecchia [34]	2012	Italy	Prospective	100 patients	HCV related liver cirrhosis	TE-LSM	0.985 for CSPH; 0.981 for SPH	0.743 for CSPH; 0.674 for SPH	NA	0.966 for CSPH; 0.959 for SPH	40 kPa for CSPH; 41.3 kPa for SPH	NA
D Attia [35]	2015	Germany	Cross-sectional	94 patients	Progressive chronic liver disease	ARFI-LSM	0.96 for CSPH; 0.94 for SPH	0.89 for CSPH; 0.89 for SPH	NA	0.929 for CSPH; 0.872 for SPH	2.32 m/s for CSPH; 2.53 m/s for SPH	NA
Bogdan Procopet [36]	2015	Spain, Canada	Prospective	88 patients	Compensated liver cirrhosis	2D-SWE (SSM)	0.90	NA	NA	0.725	22.7 kPa (rule-out); 40 kPa (rule-in)	NA
Praveen Sharma [44]	2013	India	Prospective	270 patients	Liver cirrhosis	TE-SSM	0.94 for esophageal varices	0.76 for esophageal varices	0.86 for esophageal varices	NA	40.8 kPa	NA
Xing Hu [53]	2021	China	Systematic review and meta-analysis	32 studies, 3952 patients	Chronic liver disease	TE-SSM	0.85 for CSPH; 0.84 for SPH	0.86 for CSPH; 0.84 for SPH	NA	0.92 for CSPH; 0.91 for SPH	NA	NA
Ayesha K Ahmad [56]	2019	UK	Prospective cross-sectional	25 patients	Patients with HIV without liver cirrhosis	p-SWE (SSM)	0.91	0.93	NA	0.948	25.4 kPa	NA
V Calvaruso [59]	2013	Italy	Prospective	112 patients	Compensated liver cirrhosis	TE-SSM	0.80	0.70	NA	0.820	54.0 kPa	NA
Yoshitaka Takuma [60]	2016	Japan	Prospective	60 patients	Liver cirrhosis	2D-SWE (SSM)	0.971	0.577	0.80	0.943	3.1 m/sec	NA
Christian Jansen [61]	2017	Germany, Belgium and Denmark	Prospective	158 patients	Liver cirrhosis	2D-SWE (SSM)	0.797	0.842	NA	0.84	26.3 kPa	NA
Grace Lai-Hung Wong [62]	2016	China	Cross-sectional	144 patients	HBV related liver cirrhosis	TE-SSM	NPV 0.921	PPV 0.561	NA	0.685	18.9 kPa	NA
Masashi Hirroka [63]	2011	Japan	Retrospective	60 patients	Patients with chronic liver damage	TE-SSM	0.98	0.938	0.948	NA	8.24 kPa	0.854
Johannes Vermehren [64]	2012	Germany	Prospective	166 patients	Chronic liver disease with established cirrhosis	pSWE (SSM)	0.87 for esophageal varices	0.31 for esophageal varices	NA	0.58 for esophageal varices	3.40 m/s	NA
Leonardo Rizzo [65]	2014	Italy	Retrospective	73 patients	HCV related liver cirrhosis	pSWE (SSM)	0.964	0.885	NA	0.959	3.10 m/s	NA
Christophe Cassinotto [66]	2015	France	Prospective	401 patients	Liver cirrhosis	2D-SWE (SSM)	0.94	0.36	NA	0.80	25.6 kPa for best sensitivity	NA
Laure Elkrief [67]	2015	France	Prospective	79 patients	Liver cirrhosis (mostly decompensated)	2D-SWE (SSM) and TE-SSM	0.40 with cut-off value of 34.7 kPa; 0.73 with cut-off value of 56.3 kPa	1.00 with cut-off value of 34.7 kPa; 0.67 with cut-off value of 56.3 kPa	NA	0.63 with cut-off value of 34.7 kPa; 0.64 with cut-off value of 56.3 kPa	34.7 kPa and 56.3 kPa	NA
Simona Bota [68]	2012	Romania	Retrospective	145 patients	Liver cirrhosis	pSWE (SSM)	0.967	0.211	NA	0.578	2.55 m/s	NA

SSM: spleen stiffness measurement; TE: transient elastography; CSHP: clinically significant portal hypertension; SPH: severe portal hypertension; SWE: shear-wave elastography; HBV: hepatitis B virus; HCV: hepatitis C virus; HIV: human immunodeficiency virus; AUC: area under curve.
